# Pinosylvin and Sanguinarine Combination to Enhance Antifungal Activity against *Candida albicans*

**DOI:** 10.4014/jmb.2412.12055

**Published:** 2025-05-15

**Authors:** Yaxuan Yue, Jing Liu, Chen Li, Fengfeng Chen, Cheng Yang, Bingtian Zhao

**Affiliations:** 1 Key Laboratory of Synthetic and Biological Colloids, Ministry of Education, School of Chemical and Material Engineering, Jiangnan University, Wuxi 214122, P.R. China; 2 School of Chemistry, Biology and Environment, Yuxi Normal University, Yuxi 653100, P.R. China

**Keywords:** *Candida albicans*, pinosylvin, sanguinarine, antifungal, synergy effect

## Abstract

*Candida albicans* is one of the major sources of fungal infections that can lead to life-threatening systemic infections. However, effective control of *C. albicans* remains a great challenge. Herein, this study aimed at investigating the antifungal effect of a combination of two natural compounds, pinosylvin (PIN) and sanguinarine (SAN), against *C. albicans*. In order to investigate the antifungal effect and mechanism of the combination of PIN and SAN, antimicrobial assay, time-kill assay, biofilm formation assay, cell membrane integrity assay, and reactive oxygen species (ROS) assay were performed. The results showed that the combination of PIN and SAN was more effective against *C. albicans* than PIN or SAN alone. PIN and SAN could jointly inhibit biofilm formation and thus attenuate *C. albicans* adhesion and colonization ability. PIN mainly targeting the cell membrane while SAN mainly inducing the cell to produce large amounts of ROS. Besides, PIN promoted the entry of SAN into *C. albicans*. Finally, the hemolysis experiment demonstrated that the combination of PIN and SAN is biocompatible. Taken together, the combination of PIN and SAN enhanced the antifungal effect against *C. albicans*, which has a broad application prospect in the control of *C. albicans*.

## Introduction

Compared with viral and bacterial infections, fungal infections have received less research attention and generally prove more challenging to control [1]. *Candida albicans*, a type of fungus, stands as the most common source of invasive fungal infections. These infections can give rise to both localized and systemic infections, subsequently resulting in a diverse range of disease manifestations, such as chronic mucocutaneous candidiasis, oropharyngeal candidiasis and inflammatory bowel disease [2-6]. However, frequent and prophylactic use of several antifungal agents has led to many reports of resistance [7]. For example, fluconazole, a drug predominantly employed in the treatment of fungal infections, is now confronted with the issue that *C. albicans* has developed robust resistance against it [8]. Hence, there is an immediate necessity to discover novel effective therapies and antimicrobial substances, with a focus on limiting the development of drug resistance [9]. Combination drugs are an emerging option for antimicrobial therapy. On the one hand, they may enhance antimicrobial activity resulting in shorter or reduced dosage regimens, and on the other hand to potentially diminish the speed at which pathogens acquire drug resistance [10]. Previous studies have already demonstrated that the combination of magnolol and fluconazole can act synergistically against *C. albicans* and reverse the multidrug resistance of *C. albicans* strains [11].

Currently, natural antimicrobials are a hotspot attracting a lot of attention, and it is widely recognized that they have beneficial properties and few side effects [12]. However, natural extracts are complex and it is difficult to recognize their active ingredients. Differences in the plant itself (genus, species and part of use) and the extraction method (solvent, time and temperature) will lead to variations in the content of the active ingredients, and the antimicrobial effect will be changed accordingly [13]. Hence, an increasing number of scholars have begun to study the antimicrobial properties of natural pure compounds. The studied ones are, but not limited to, plant flavonoids, polyphenols, and alkaloids, which generally possess a variety of biological activities such as anti-inflammatory, anticancer, and antioxidant in addition to antimicrobial [14-16]. Nevertheless, many studies have focused only on antimicrobial capacity, and few researchers have investigated the synergistic effects of different proportions of drugs as well as the underlying antimicrobial mechanisms between drugs.

Pinosylvin (PIN), which is a naturally sourced stilbene, has a characteristic occurrence mainly in the heartwood and knotwood of trees within the *Pinus* family [17]. Sanguinarine (SAN), categorized as an isoquinoline alkaloid, occurs naturally in a number of traditional herbs, with *Chelidonium*, *Corydalis* and *Macleaya* being among them [18]. They are plant secondary metabolites with excellent biological activities including antifungal activity [17, 18]. However, the antifungal effect and underlying antifungal mechanism of the combination of PIN and SAN have not been previously reported. In this study, we focused on the antifungal synergistic potential of the combination of PIN and SAN against *C. albicans*, and it was found that the antifungal effect was enhanced when they were used in combination, so we further explored the antifungal mechanism of the combination of PIN and SAN. Overall, this work provides a rationale for the combination of PIN and SAN in controlling *C. albicans*.

## Materials and Methods

### Strain and Culture Condition

*C. albicans* (BNCC 336485) was purchased from BeNa Culture Collection Center (China), and incubated in yeast extract peptone dextrose (YPD) medium at 30°C. The culture to logarithmic growth phase was transferred to centrifuge tubes, centrifuged, and the microorganisms were redispersed in liquid medium after removal of the supernatant, and *C. albicans* suspension was obtained by resuspension twice in this manner and kept for reserve.

### Chemicals

PIN (CAS: 22139-77-1) and SAN (CAS: 2447-54-3) were obtained from Shanghai Aladdin Biochemical Science and Technology Co., Ltd., with purity ≥ 97%. SYTO 9 and PI were purchased from Thermo Fisher Scientific (USA). The reactive oxygen species (ROS) assay kit was purchased from Beyotime (China).

### Determination of the Minimum Inhibitory Concentration (MIC) and the Minimum Fungicidal Concentration (MFC)

Each sample was diluted two-fold in a microplate, and then the prepared *C. albicans* suspension was added so that each well contained 10^6^ CFU/ml of *C. albicans* cells. Place the 96-well plate in an incubator at 30°C for 24 h. The MIC was determined as the lowest concentration of the samples without the growth of visible fungi. After that, dilutions of the test suspensions in the above wells were further incubated for 48 h using YPD agar medium to determine MFC. The lowest concentration of the samples at which less than 0.1% of the colonies grew was taken as the MFC. Furthermore, MFC/MIC ratios were also computed. Three parallel experiments were done each time.

### Checkerboard Assay and Drug Synergy Analysis

In a 96-well plate, PIN was added to the first column and then diluted along the horizontal coordinate. Subsequently, a twofold serial dilution of SAN was added to each row separately. The prepared *C. albicans* suspension was then added so that each well contained 10^6^ CFU/ml of *C. albicans*. The microplate was incubated at 30°C for 24 h. The optical density (OD) at 600 nm was detected by a microplate reader (SpectraMax M2) [19]. The MIC was defined as the lowest concentration at which no turbidity could be observed (with an OD_600_ < 0.1). The fractional inhibitory concentration (FIC) was calculated as the following formula:

FIC index = MIC_PS_/MIC_P_+ MIC_SP_/MIC_S_

MIC_P_ is the MIC of PIN alone, MIC_PS_ is the MIC of PIN in combination with SAN, MIC_S_ is the MIC of SAN alone, MIC_SP_ is the MIC of SAN in combination with PIN. FIC ≤ 0.5 suggests a synergistic inter-action, while a range of 0.5 to 1.0 indicates an additive interaction. A FIC value of 1.0 to 4.0 indicates an indifference effect, whereas a FIC > 4.0 suggests an antagonistic interaction [20]. Drug synergy or antagonism was analyzed by Combenefit software with Bliss independence criterion model [21, 22].

### Time-Kill Assay

Tubes were filled with different concentrations of samples and diluted *C. albicans* suspension. Incubation was carried out in a shaker at 30°C. At the appointed time (within 24 h), dilutions of the test suspensions in the above tubes were further incubated for 48 h using YPD agar medium. The cell density of each sample was estimated from the colony counts [23].

### Scanning Electron Microscopy

*C. albicans* cells (10^7^ CFU/ml) were cultured with PIN, SAN, the combination, and control, respectively. After 24 h, the cells were obtained and rinsed two times with phosphate buffered saline (PBS, pH = 7), freeze-dried, and then fixed on a carrier for gold sputtering in an argon environment. The cells were observed with a scanning electron microscope (SEM, S-4800) with the magnification of 5,000 × and 10,000 ×.

### Biofilm Formation Experiment

The obtained bacterial solution was diluted with liquid medium to 2 × 10^6^ CFU/ml. 100 μl of diluted *C. albicans* suspension and 100 μl of sample were added to each well of a 96-well plate, and the total volume was 200 μl. And then, the seeded microplate was incubated at 30°C for 24 h. The biofilm was gently washed with sterile PBS for 3 times, and then 2.5% glutaraldehyde was added to fix it for 2 h. The sample was immersed in 0.1% crystal violet solution for 15 min to stain. Afterward, it was carefully rinsed with PBS several times to thoroughly wash away the extra crystal violet. Then, 150 μl of anhydrous ethanol was introduced for the purpose of destaining. The formation of biofilm was reflected by measuring the OD_590_ in 100 μl of the supernatant [24]. The biofilm formation rate was calculated as the formula:

$$\text{Biofil formation rate}\left(\%\right)=\frac{OD_{sample}-OD_{blank}}{OD_{control}-OD_{blank}}\times 100\%$$

### Cell Membrane Integrity Assay

Centrifuge tubes were filled with diluted *C. albicans* suspension and samples. 75% ethanol and PBS were served as the positive and negative controls, respectively. The samples were incubated in a shaker at 30°C for 24 h. The supernatant was centrifuged and discarded, and a mixed dye solution of 5 μM SYTO 9 (green) and 30 μM PI (red) was added, and the excess dye was washed away with PBS after 15 min. SYTO 9 and PI were observed by a confocal laser scanning microscope (CLSM, TCS SP8) at excitation/emission wavelengths of 488/525 nm and 535/617 nm, respectively. The fluorescent dye of SYTO 9 (membrane-permeant) and PI (membrane-impermeant) were used to assess cell membrane integrity, the percentage of membrane-damaged cells was statistically derived by ImageJ software.

### Detection of Intracellular ROS Production

Centrifuge tubes were filled with *C. albicans* suspension and samples, then incubated at 30°C for 3 h. The positive control was treated with 1.5 mM H_2_O_2_ and the negative control was the corresponding solvent for the sample group. Subsequently, the organisms were stained with 10 μM DCFH-DA for 20 min, collected by centrifugation and washed with PBS. The fluorescence was observed with a CLSM at excitation/emission wavelengths of 488/ 525 nm [25]. Total fluorescence intensity was analyzed semi-quantitatively by ImageJ software.

### SAN Autofluorescence Assay

Briefly, *C. albicans* cells were treated with SAN alone or in combination with PIN for 24 h and then washed before intracellular SAN autofluorescence was detected by a CLSM under 488 nm excitation and 600 nm emission. ImageJ software was applied to semi-quantitatively analyze the total fluorescence intensity of SAN autofluorescence.

### Hemolysis Assay

The 4% rabbit red blood cells (RBCs) were subjected to washing with PBS until the supernatant achieved clarity. The 500 μl rabbit RBCs and 500 μl of sample were added in eppendorf tubes. 0.1% triton X-100, DMSO solution, and PBS were served as the positive, negative, and blank controls, respectively. After being incubated at 37°C for 1 h, the mixture was centrifuged at 1,000 g for 5 min. Subsequently, 200 μl of the supernatant was transferred to a 96-well plate. Hemoglobin release was measured by monitoring OD at 576 nm [14]. All experiments were performed in triplicate. The hemolysis rate was calculated as the formula:

$$\text{Hemolysis rate}\left(\%\right)=\frac{OD_{sample}-OD_{blank}}{OD_{positive}-OD_{blank}}\times 100\%$$

### Statistical Analysis

Data are presented as mean ± SD. Statistical analysis was performed using one-way analysis of variance (ANOVA) test. The data analysis was conducted using SPSS statistical software (version 25.0), with the statistical significance level set at *p* < 0.05. *p < 0.05; **p < 0.01; ***p < 0.001.

## Results

### Antifungal Activities of PIN and SAN against *C. albicans*

As shown in Table 1, both PIN and SAN inhibited *C. albicans* with MIC values of 50 μg/ml. Additionally, the MFC value of PIN against *C. albicans* was 100 μg/ml, and the MFC/MIC ratio was equal to 2. Whereas the MFC value of SAN (> 4 × MIC) was not determined.

### The Interaction between PIN and SAN against *C. albicans*

Samples with different concentrations of PIN and SAN were recorded as P-nMIC and S-nMIC, respectively, based on their MIC values. *e.g.*, 1 × MIC PIN was noted as P-1MIC, 1/2 × MIC PIN was noted as P-1/2MIC, and so on; similarly, 1 × MIC SAN was labeled as S-1MIC, 1/2 × MIC SAN was labeled as S-1/2MIC, and so on; and the combination of 1 × MIC PIN and 1 × MIC SAN was recorded as P-1MIC+S-1MIC, the combination of 1/2 × MIC PIN and 1/2 × MIC SAN was recorded as P-1/2MIC+S-1/2MIC, and so on.

**Fig. 1:**
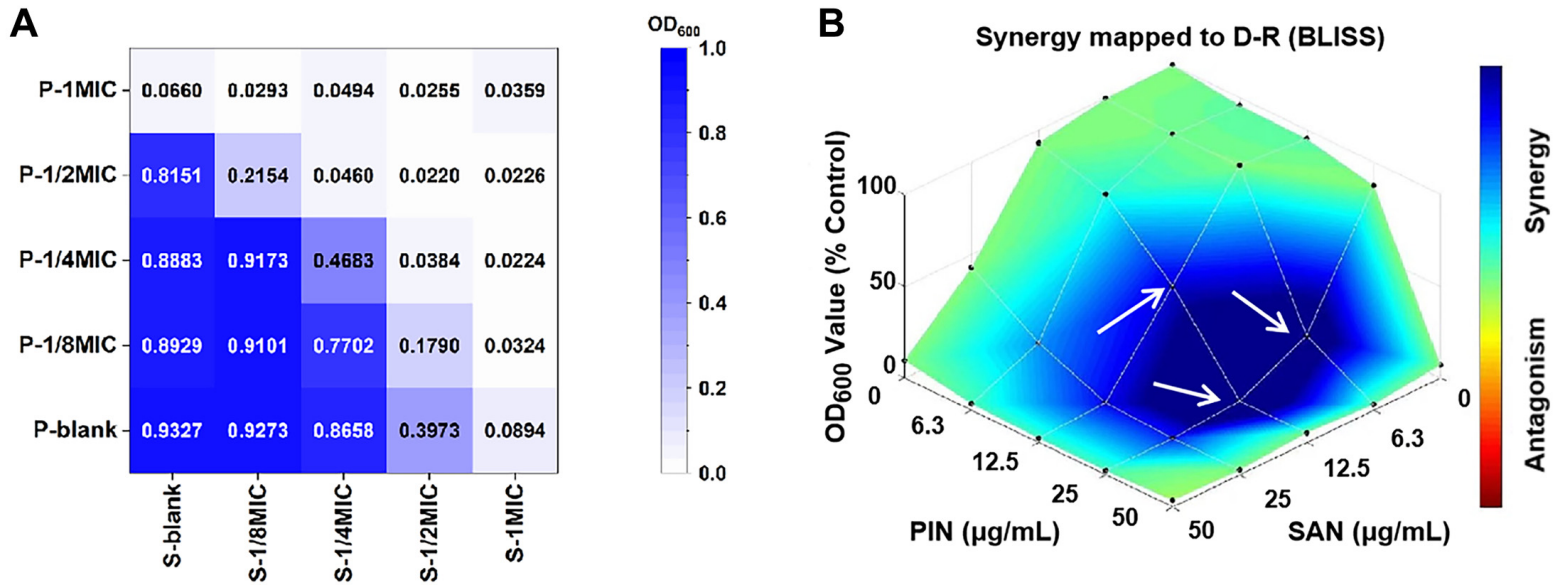
The interaction between PIN and SAN against *C. albicans*. (**A**) The checkerboard plot of the antimicrobial activity of the combination of PIN and SAN on *C. albicans*. (**B**) Synergy distribution mapped to the dose-response surface by the Bliss independence model. White arrows point to the areas where the strongest synergies occur.

The checkerboard method was used to assess the interaction of PIN and SAN. As illustrated in Fig. 1A, P-1/2MIC completely inhibited *C. albicans* when combined with S-1/4MIC, indicating an additive effect against *C. albicans* based on the FIC index, with a FIC value of 0.75.

The OD value at 600 nm reflects the amount of *C. albicans*. Additionally, the absorbance of P-1/2MIC+S-1/2MIC (OD_600_ = 0.022) was lower than that of P-1MIC (OD_600_ = 0.066) and S-1MIC (OD_600_ = 0.089). This indicated that the antimicrobial effect of the combination was stronger than that of either PIN or SAN alone at the same concentration.

The drug synergism or antagonism was determined with the most commonly used models, Bliss independence criterion model, utilizing Combenefit software, as shown in Fig. 1B where darker blue color means stronger synergistic effects, and the results showed that the combinations were strongly synergistic at three PIN:SAN mass ratios (2:1, 4:1 and 1:1), with the strongest synergistic effect being seen when P-1/2MIC was combined with S-1/4MIC, followed by P-1/2MIC+S-1/8MIC and P-1/4MIC+S-1/4MIC. In order to facilitate the comparison of the antimicrobial properties of individual PIN/SAN and the combination, a PIN:SAN ratio of 1:1 was used in subsequent experiments.

### Time-Kill Evaluation on *C. albicans*

**Fig. 2:**
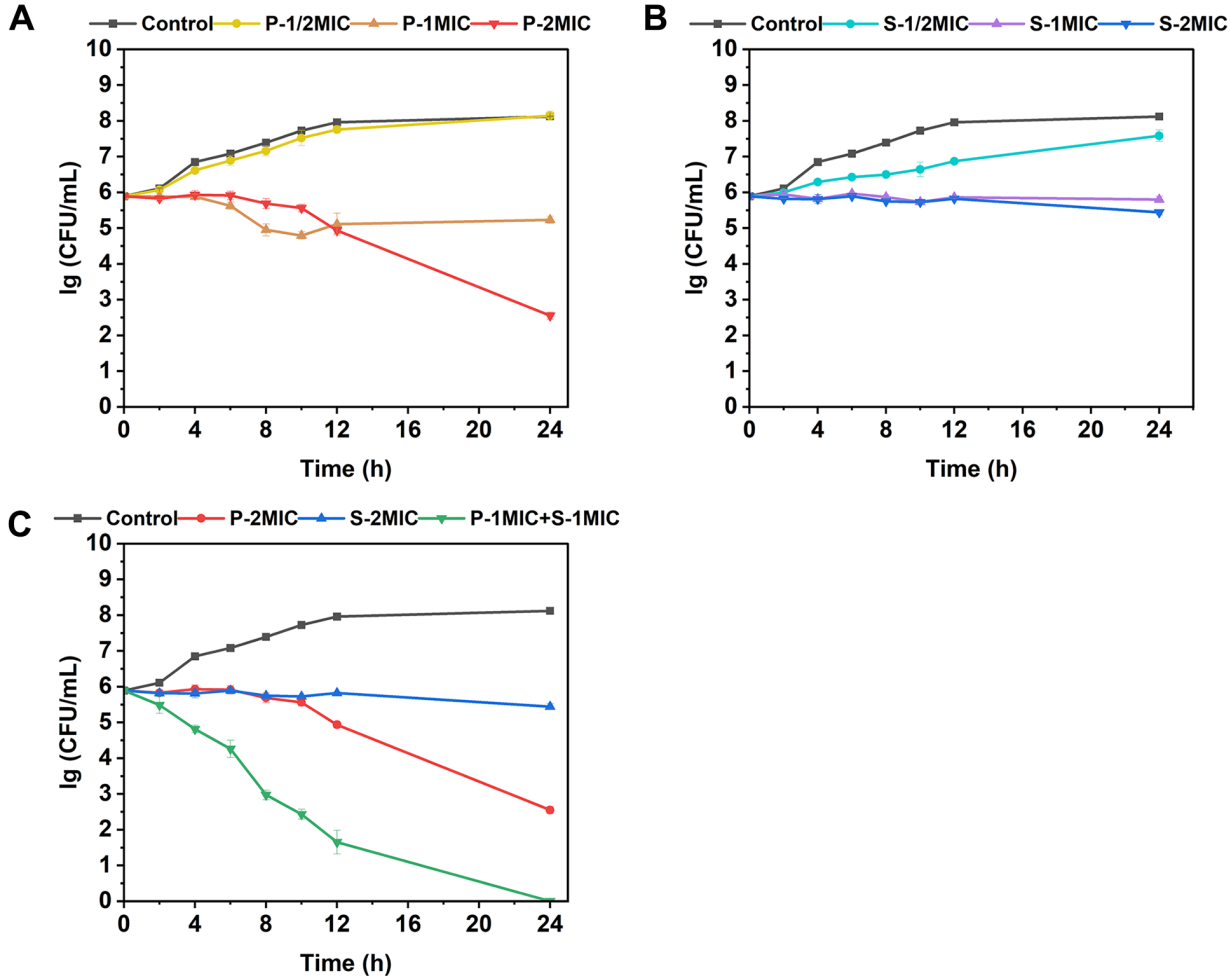
Time-kill curves of *C. albicans* cells treated with PIN, SAN, the combination, and control. (**A**) Treated with different concentrations of PIN. (**B**) Treated with different concentrations of SAN. (**C**) Treated with P-1MIC+S-1MIC and with PIN or SAN at equivalent concentrations.

By examining the time-kill curves of *C. albicans* cells treated with PIN, SAN, the combination, and control, it was found that the number of cells in the control (no antimicrobials) reached about 10^8^ CFU/ml at 24 h, while the number of cells treated with different concentrations of PIN or SAN was lower than the control (Fig. 2A and 2B). Besides, time-kill curves showed strong concentration-dependent antifungal activity for PIN, which means that the antifungal activity of PIN was closely related to the concentration. It can be seen in the Fig. 2A that PIN had almost no antifungal effect at 1/2 × MIC concentration, completely inhibited the growth of *C. albicans* at 1 × MIC concentration, and killed 99.9% of cells at 2 × MIC concentration.

Interestingly, the fungicidal activity of P-1MIC+S-1MIC was significantly stronger than that of P-2MIC and S-2MIC (Fig. 2C): after 24 h of treatment, there were no colonies in the coated plates of P-1MIC+S-1MIC, which indicated that it could completely kill *C. albicans* cells, whereas, for PIN and SAN alone, the number of cells was slightly reduced for the S-2MIC treatment and by only three orders of magnitude for the P-2MIC compared to the initial cell number. Moreover, the combination of agents showed significant fungicidal effect after 2 h, however, PIN alone began to show fungicidal effect only after 10 h of treatment and SAN alone only inhibited growth and reproduction. The fungicidal efficiency was increased after compounding.

### Morphological Changes of *C. albicans* in the presence of PIN and SAN alone or in Combination

**Fig. 3:**
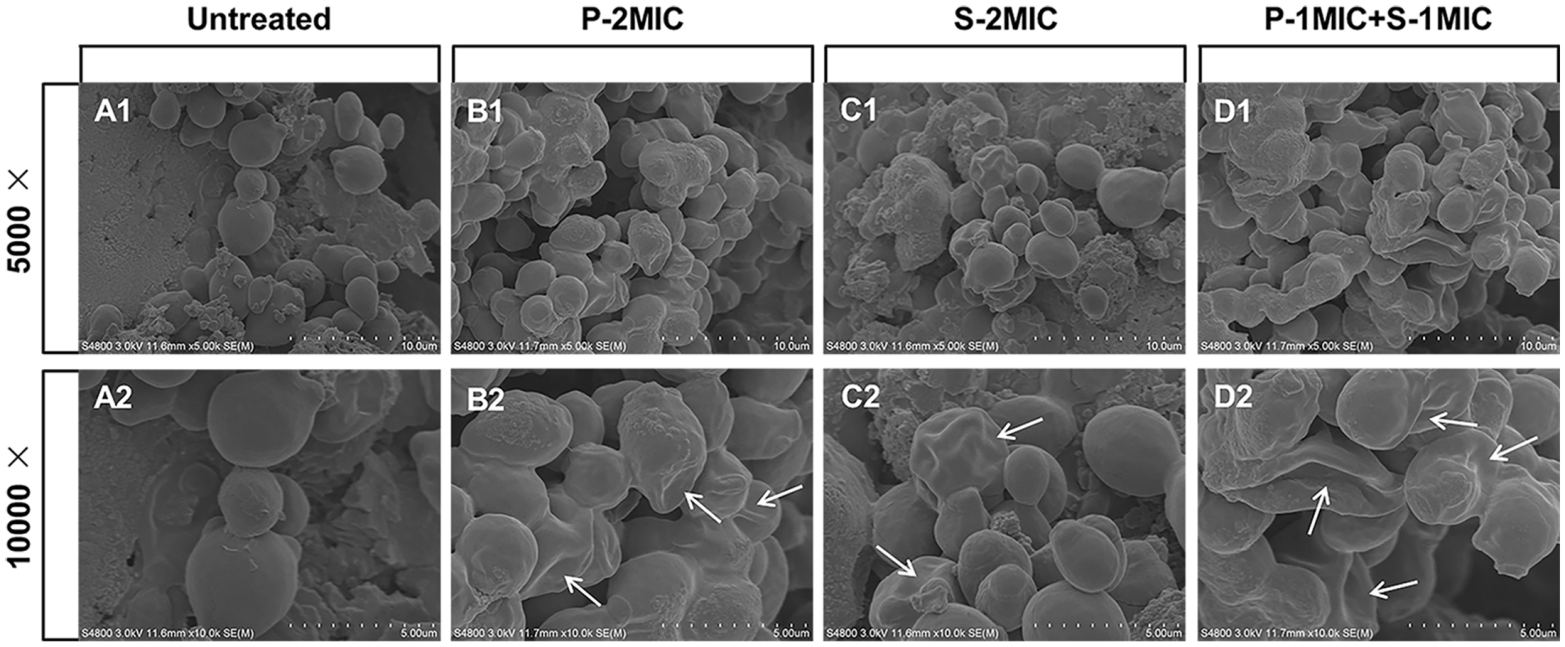
The morphology of *C. albicans* by SEM. (**A1**, and **A2**) Untreated; (**B1**, and **B2**) Treated with P-2MIC; (**C1**, and **C2**) Treated with S-2MIC; (**D1**, and **D2**) Treated with P-1MIC+S-1MIC. The arrows indicate significant changes in *C. albicans* morphology.

In the morphological observation via SEM (Fig. 3), the surface of *C. albicans* cells was collapsed after the treatment of PIN (Fig. 3B1 and 3B2); the cells were slightly crumpled after the treatment of SAN, and some of them could basically keep the original morphology (Fig. 3C1 and 3C2); and the combination of PIN and SAN could make them seriously collapsed and distorted under the equivalent concentration (Fig. 3D1 and 3D2). Comparison of the smooth surface and full morphology of normal cells (Fig. 3A1 and 3A2), PIN and SAN could change the morphology of *C. albicans*, and the combination changed it even worse.

### PIN and SAN Jointly Inhibit *C. albicans* Biofilm Formation

**Fig. 4:**
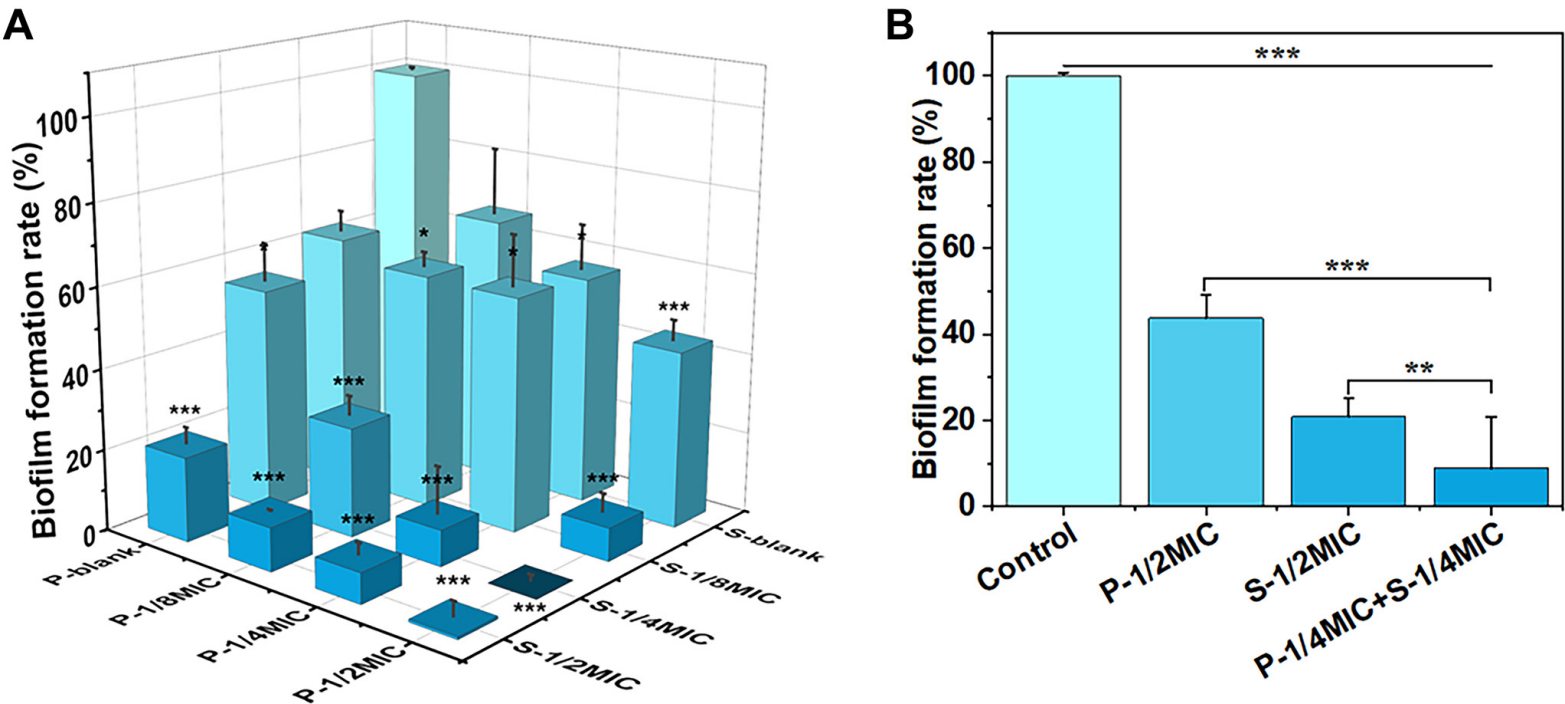
Biofilm formation of *C. albicans* cells after being treated by PIN, SAN, the combination, and control. (**A**) The three-dimensional plot of the effects of different concentrations of PIN, SAN, and combinations on biofilms. (**B**) Comparison of the effects of PIN, SAN, and the combination on biofilms at equivalent concentrations. Data are presented as mean ± SD (*n* = 3). *p < 0.05, **p < 0.01 and ***p < 0.001 indicate significant differences.

Biofilm formation of *C. albicans* cells was determined by crystal violet staining. Compared with the control, the rate of biofilm formation was reduced in all treatment groups and decreased with increasing drug concentration (Fig. 4A). When PIN and SAN were used alone against *C. albicans*, both had significant inhibitory effects on *C. albicans* biofilm at concentrations up to 1/2 × MIC, with biofilm formation rates of 43.78% and 20.88%, respectively. Surprisingly, the combination of P-1/4MIC+S-1/4MIC showed noteworthy inhibition of *C. albicans* biofilm, and the biofilm formation rate was as low as 8.95%, which was obviously lower than that of PIN (P-1/ 2MIC) or SAN (S-1/2MIC) at the equivalent concentration (Fig. 4B). It is implied that PIN and SAN could jointly inhibit *C. albicans* biofilm formation and that the combined use is significantly more effective than their individual use.

### PIN in the Combination Targets the Cell Membrane of *C. albicans*

**Fig. 5:**
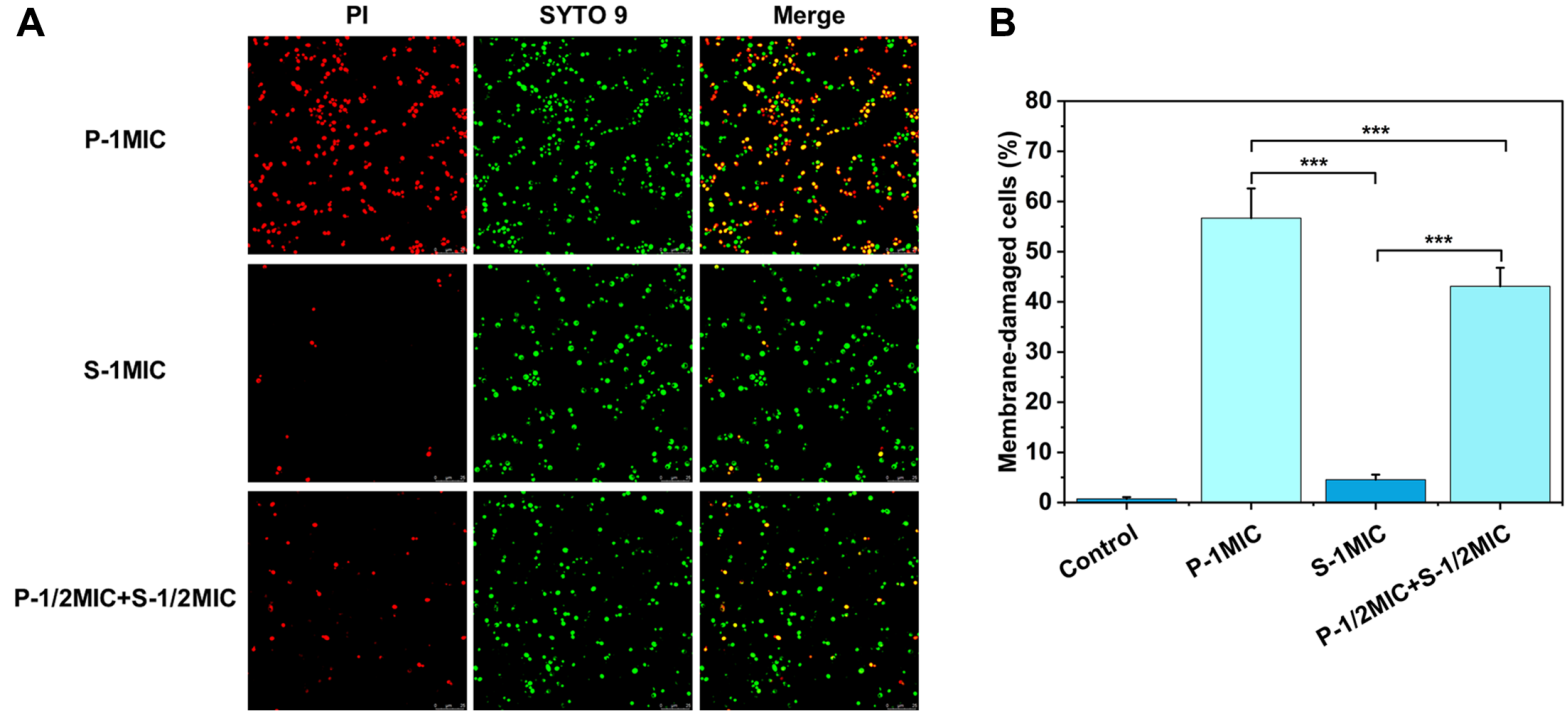
Cell membrane damage status of *C. albicans* after treatment with PIN, SAN or the combination. (**A**) The CLSM images of *C. albicans* stained by PI and SYTO 9. PI (red) staining shows the disruptive effect of the sample on cell membrane integrity. SYTO 9 (green) staining marks the number of cells. (**B**) The plot of the percentage of membrane-damaged cells. Data are presented as mean ± SD (*n* = 3). *p < 0.05, **p < 0.01 and ***p < 0.001 indicate significant differences.

Cells with damaged cell membranes can be stained by PI, which cannot penetrate living cells with intact cell membranes and only enters cells with damaged cell membranes. SYTO 9 is a permeable dye that penetrates all cell membranes. 56.69% of PI*N*-treated cells with damaged membranes were stained by PI, but only 4.55% of SA*N*-treated cells were stained, and the percentage of membrane-damaged cells treated with the combination was intermediate at 43.08% (Fig. 5), indicating that PIN had a stronger disruptive effect on cell membranes, whereas SAN had a weaker effect on cell membranes.

### The Combinational Use of PIN and SAN Boosts Intracellular ROS Production

**Fig. 6:**
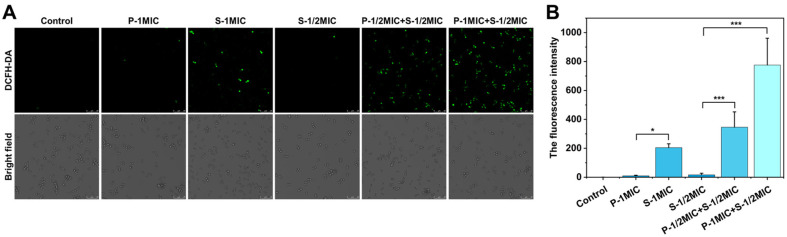
The intracellular ROS production in *C. albicans*. (**A**) The CLSM images of *C. albicans* stained by DCFH-DA, green fluorescence reflects intracellular ROS. (**B**) The total fluorescence intensity graph was obtained by ImageJ software analysis of the CLSM images. Data are presented as mean ± SD (*n* = 3). *p < 0.05, **p < 0.01 and ***p < 0.001 indicate significant differences.

To evaluate the underlying mechanism for antifungal efficacy, we examined the intracellular ROS, which was stained by DCFH-DA as shown in Fig. 6. Significant ROS production was observed in *C. albicans* cells at S-1MIC, while PIN had almost no effect on intracellular ROS production under the concentration of 1 × MIC. Large amounts of intracellular ROS were observed at equivalent concentrations of the combination (P-1/2MIC+S-1/ 2MIC), exceeding both S-1MIC and P-1MIC.

Under the same concentration of 25 μg/ml SAN (S-1/2MIC), different concentrations of PIN were added. The result was observed that intracellular ROS gradually increased with the increase in the concentration of PIN, which indicated that PIN enhanced the ability of SAN to stimulate intracellular ROS production.

### PIN Promotes Endocytosis of SAN by *C. albicans*

**Fig. 7:**
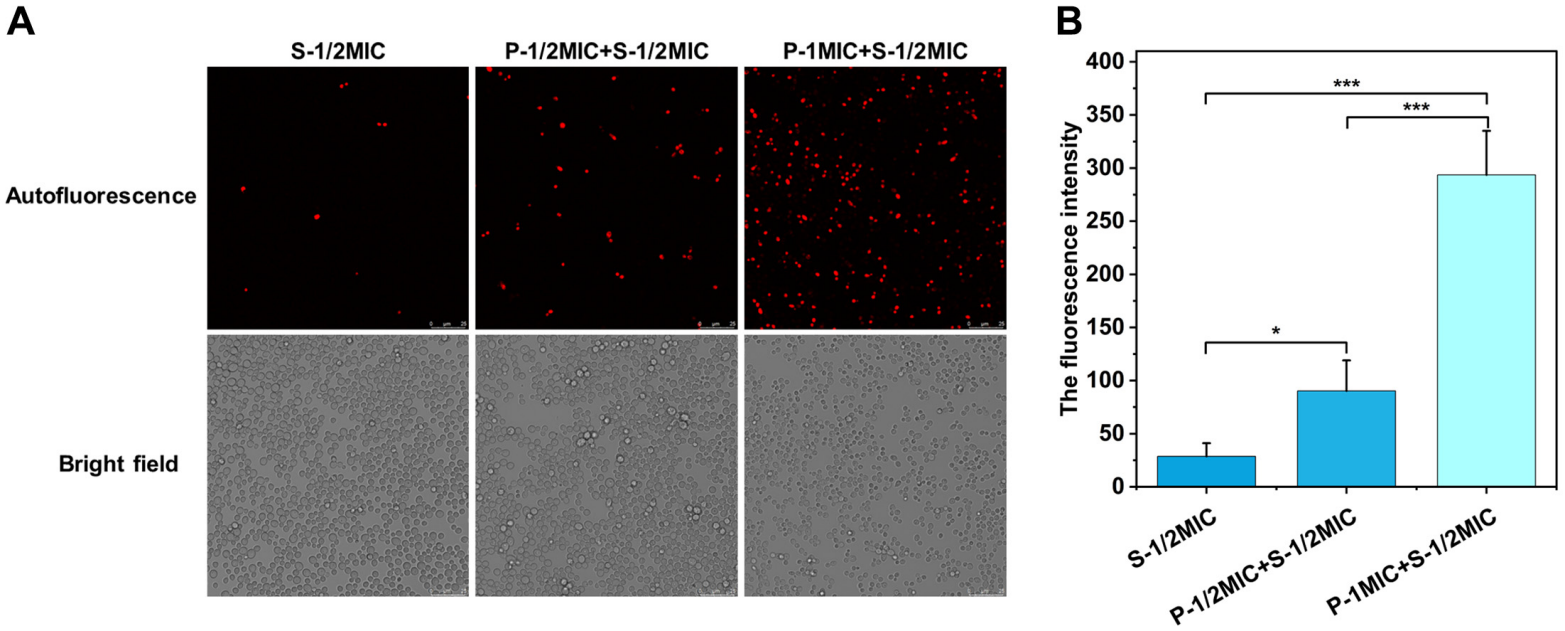
Effect of different concentrations of PIN on endocytosis of SAN by *C. albicans*. (**A**) Autofluorescence pictures of intracellular SAN (red) observed in *C. albicans*. (**B**) The total fluorescence intensity graph. Data are presented as mean ± SD (*n* = 3). *p < 0.05, **p < 0.01 and ***p < 0.001 indicate significant differences.

Endocytosis of SAN by *C. albicans* was determined through autofluorescence of SAN using CLSM as shown in Fig. 7. SAN has autofluorescence around 600 nm. Taking advantage of this feature, the autofluorescence was observed in *C. albicans* after treatment with SAN alone or in combination with PIN, which reflects the enrichment of SAN in the cells. Under the same concentration of 25 μg/ml SAN (S-1/2MIC), different concentrations of PIN were added. The result was observed that intracellular autofluorescence of SAN gradually increased with the increase in the concentration of PIN, which indicated that the endocytosis of SAN by the cells gradually increased with the increase of PIN concentration. This is consistent with the pattern of ROS assay.

### The Effect of PIN and SAN alone or in Combination on Hemolysis

**Fig. 8:**
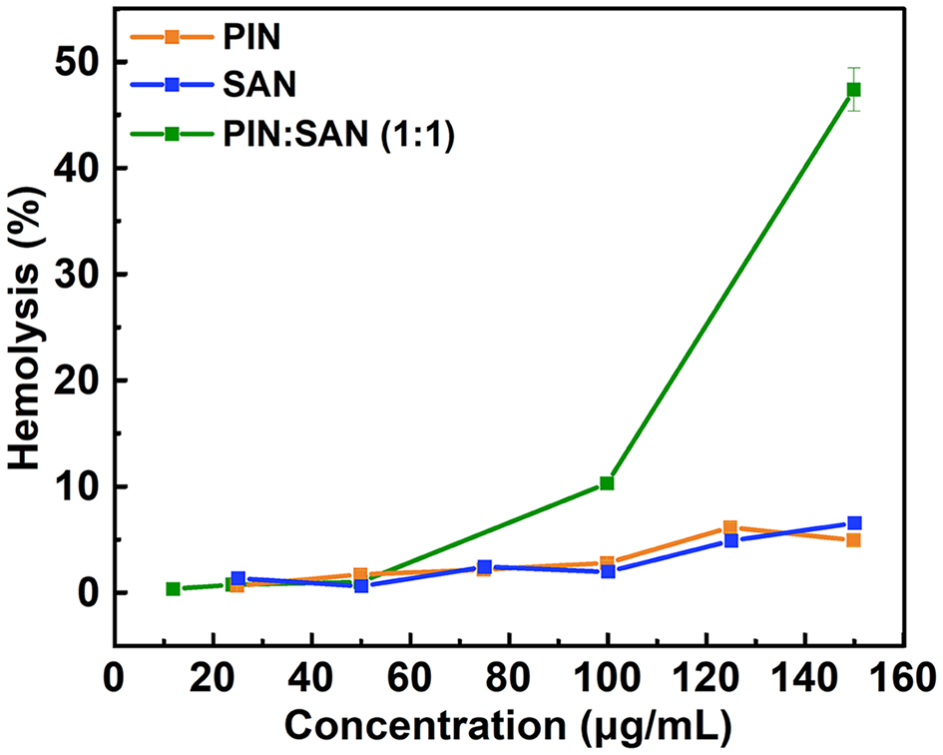
Hemolysis rates of different concentrations of PIN, SAN and the combination to reflect their biocompatibility.

Evaluation of biocompatibility by hemolysis assay. As shown in Fig. 8, no hemolysis of erythrocytes was observed at concentrations up to 150 μg/ml for PIN/SAN alone, and the hemolysis rate was less than 10%. In contrast, the combination (PIN:SAN mass ratio of 1:1) was more prone to hemolysis. However, at a concentration of 100 μg/ml, the hemolysis rate reached only 10%. Moreover, this concentration proved powerful enough to eradicate all the tested *C. albicans*, as evidenced by the time-kill curve of P-1MIC+S-1MIC (Fig. 2C). In comparison, upon reaching a concentration of 150 μg/ml for the combination, the hemolysis rate approximated 45%.

## Discussion

Lee *et al*. reported the antimicrobial effect of PIN with a MIC of 62.5 μg/ml against *C. albicans*, which is comparable to the result of 50 μg/ml in this study [26]. The MFC value of PIN was also measured to be 100 μg/ml. The MFC/MIC of PIN was equal to 2, that was less than 4 indicating that it has fungicidal activity. Whereas the MFC of SAN (> 4 × MIC) was not determined, the ratio of MFC/MIC was higher than 4, indicating that it is difficult to achieve a microbicidal dose, classifying the compound as a fungistatic agent [27].

Välimaa *et al*. demonstrated the broad-spectrum antimicrobial activity of PIN, showing antimicrobial effects against several bacteria, as well as fungi (*Saccharomyces cerevisiae*, *Aspergillus fumigatus*, and *Penicillium brevicompactum*) [17]. Although PIN has strong antimicrobial activity, its antimicrobial mechanism against *C. albicans* has not been previously reported, l*et al*one its complexation studies with other compounds. Relatively more research has been done on SAN. However, there is a gap in the research on its combination with other antimicrobial agents against *C. albicans*.

In the present study, the interaction between PIN and SAN was first assessed by the checkerboard assay. According to the definition of FIC, there is an additive interaction between PIN and SAN. Based on the synergy analysis, the combination of PIN and SAN enhanced the inhibition of *C. albicans* relative to PIN or SAN alone. The relationship between time and the extent to which *C. albicans* was killed was then further assessed by time-kill curves, which showed that PIN in combination with SAN killed *C. albicans* more rapidly and more completely. Briefly, these results indicated that the combination of PIN and SAN was more effective against *C. albicans* than PIN or SAN alone, enhancing the inhibitory effect at lower concentrations (1 × MIC) and increasing the fungicidal ability at higher concentrations (2 × MIC).

In order to find out the reason for the combination possessing enhanced antifungal ability against *C. albicans*, the antifungal mechanisms of PIN and SAN alone or in combination were then investigated. It was found that the antimicrobial mechanism of PIN against *C. albicans* is inhibition of biofilm formation and disruption of cell membrane integrity, while SAN has a strong ability to inhibit biofilm formation and ROS production. The combination collectively inhibited biofilm formation on the one hand, and on the other hand, PIN promoted the entry of SAN into *C. albicans* as evidenced by SAN autofluorescence assay, which may further accelerate apoptosis.

Finally, biocompatibility was evaluated by hemolysis experiments. Reagents showing hemolysis values below 10% are typically classified as non-hemolytic, whereas reagents with hemolysis values exceeding 25% present a risk of hemolysis [28]. Based on this, both PIN/SAN alone and their combination remain biocompatible at effective concentrations. In this study, only the hemolysis experiment was conducted as an indicator for safety assessment, but various toxicological data are yet to be further evaluated.

Additionally, conventional theory suggests that bactericidal drugs in combination with bacteriostatic drugs tend to show antagonistic effects, probably because bactericidal drugs are most effective against actively growing cells while bacteriostatic drugs stagnate bacterial growth, thus depriving bactericidal drugs of their killing power [29-31]. Ocampo *et al*. investigated the potential drug-drug interactions of six bactericide and bacteriostat combinations with different mechanisms of action against *E. coli*, showing that the addition of a bacteriostat in the presence of a bactericide led to a decrease in the killing rate, in addition to investigating 204 combinations of antibiotics by high-throughput methodology, of which 61% showed antagonistic effects [32]. The combination of PIN and SAN reported in this paper may be an exception to this rule. The reason for obtaining such synergic results may be that different mechanisms (surface/interior) and different types (stilbene/isoquinoline alkaloid) of compounds were initially selected in this study. Although the above results have been achieved, the effectiveness of this combination therapy for other antifungal drugs needs to be further investigated.

## Conclusion

Concisely, the combination of PIN and SAN has a broad application prospect in the control of *C. albicans* due to the good biocompatibility and potential synergistic antifungal effect of the combination. Its main synergistic functions stem from the disruption of the fungal membrane by PIN and the generation of intracellular ROS stimulated by SAN. This combination approach may provide a new reference for later studies of similar combination systems in the antibacterial and antiseptic fields.

## Figures and Tables

**Table 1:** The MIC and MFC values of compounds against *C. albicans*.

Compounds	MIC (μg/ml)	MFC (μg/ml)
PIN	50	100
SAN	50	ND^1^

^1^ND = Not determined.

## References

[1] Morard M, Pérez-Través L, Perpiñá C, Lairón-Peris M, Collado MC, Pérez-Torrado R (2023). Comparative genomics of infective *Saccharomyces cerevisiae* strains reveals their food origin. Sci. Rep..

[2] Sprague JL, Kasper L, Hube B (2022). From intestinal colonization to systemic infections: *Candida albicans* translocation and dissemination. Gut Microbes.

[3] Ho J, Camilli G, Griffiths JS, Richardson JP, Kichik N, Naglik JR (2021). *Candida albicans* and candidalysin in inflammatory disorders and cancer. Immunology.

[4] Kashem SW, Kaplan DH (2016). Skin immunity to *Candida albicans*. Trends Immunol..

[5] Salvatori O, Puri S, Tati S, Edgerton M (2016). Innate immunity and saliva in *Candida albicans*-mediated oral diseases. J. Dent. Res..

[6] Peng Z, Tang J (2021). Intestinal infection of *Candida albicans*: preventing the formation of biofilm by *C. albicans* and protecting the intestinal epithelial barrier. Front. Microbiol..

[7] Lee Y, Puumala E, Robbins N, Cowen LE (2021). Antifungal drug resistance: molecular mechanisms in *Candida albicans* and beyond. Chem. Rev..

[8] Bédard C, Gagnon-Arsenault I, Boisvert J, Plante S, Dubé AK, Pageau A (2024). Most azole resistance mutations in the *Candida albicans* drug target confer cross-resistance without intrinsic fitness cost. Nat. Microbiol..

[9] Simões NG, Bettencourt AF, Monge N, Ribeiro IAC (2017). Novel antibacterial agents: an emergent need to win the battle against infections. Mini-Rev. Med. Chem..

[10] Cottarel G, Wierzbowski J (2007). Combination drugs, an emerging option for antibacterial therapy. Trends Biotechnol..

[11] Sun LM, Liao K, Liang S, Yu PH, Wang DY (2015). Synergistic activity of magnolol with azoles and its possible antifungal mechanism against *Candida albicans*. J. Appl. Microbiol..

[12] Dong HY, Xu Y, Zhang QQ, Li H, Chen LX (2024). Activity and safety evaluation of natural preservatives. Food Res. Int..

[13] Camaioni L, Ustyanowski B, Buisine M, Lambert D, Sendid B, Billamboz M (2023). Natural compounds with antifungal properties against *Candida albicans* and identification of hinokitiol as a promising antifungal drug. Antibiotics.

[14] Song M, Liu Y, Li T, Liu X, Hao Z, Ding S (2021). Plant natural flavonoids against multidrug resistant pathogens. Adv. Sci..

[15] Bostanghadiri N, Pormohammad A, Chirani AS, Pouriran R, Erfanimanesh S, Hashemi A (2017). Comprehensive review on the antimicrobial potency of the plant polyphenol resveratrol. Biomed. Pharmacother..

[16] Zhao ZM, Shang XF, Lawoe RK, Liu YQ, Zhou R, Sun Y (2019). Anti-phytopathogenic activity and the possible mechanisms of action of isoquinoline alkaloid sanguinarine. Pestic. Biochem. Phys..

[17] Välimaa AL, Honkalampi-Hämäläinen U, Pietarinen S, Willför S, Holmbom B, von Wright A (2007). Antimicrobial and cytotoxic knotwood extracts and related pure compounds and their effects on food-associated microorganisms. Int. J. Food Microbiol..

[18] Huang LJ, Lan JX, Wang JH, Huang H, Lu K, Zhou ZN (2024). Bioactivity and mechanism of action of sanguinarine and its derivatives in the past 10 years. Biomed. Pharmacother..

[19] Song M, Liu Y, Huang X, Ding S, Wang Y, Shen J (2020). A broad-spectrum antibiotic adjuvant reverses multidrug-resistant gram-negative pathogens. Nat. Microbiol..

[20] Swetha TK, Vikraman A, Nithya C, Hari Prasath N, Pandian SK (2020). Synergistic antimicrobial combination of carvacrol and thymol impairs single and mixed-species biofilms of *Candida albicans* and *Staphylococcus epidermidis*. Biofouling.

[21] Lu C, Zhang N, Kou S, Gao L, Peng B, Dai Y (2022). Sanguinarine synergistically potentiates aminoglycoside-mediated bacterial killing. Microb. Biotechnol..

[22] Di Veroli GY, Fornari C, Wang D, Mollard S, Bramhall JL, Richards FM (2016). Combenefit: an interactive platform for the analysis and visualization of drug combinations. Bioinformatics.

[23] Yang L, Wang X, Ma Z, Sui Y, Liu X (2024). Fangchinoline inhibits growth and biofilm of *Candida albicans* by inducing ROS overproduction. J. Cell Mol. Med..

[24] Maione A, Imparato M, Buonanno A, Carraturo F, Schettino A, Schettino MT (2023). Anti-biofilm activity of phenyllactic acid against clinical isolates of fluconazole-resistant *Candida albicans*. J. Fungi.

[25] Sun L, Liao K, Hang C, Wang D (2017). Honokiol induces reactive oxygen species-mediated apoptosis in *Candida albicans* through mitochondrial dysfunction. PLoS One.

[26] Lee SK, Lee HJ, Min HY, Park EJ, Lee KM, Ahn YH (2005). Antibacterial and antifungal activity of pinosylvin, a constituent of pine. Fitoterapia.

[27] Nouioura G, El Fadili M, El Hachlafi N, Abuelizz HA, Elidrissi AE, Ferioun M (2024). *Petroselinum crispum* L., essential oil as promising source of bioactive compounds, antioxidant, antimicrobial activities: *In vitro* and in silico predictions. Heliyon.

[28] Amin K, Dannenfelser RM (2006). *In vitro* hemolysis: guidance for the pharmaceutical scientist. J. Pharm. Sci..

[29] Jawetz E, Gunnison JB (1953). Antibiotic synergism and antagonism; an assessment of the problem. Pharmacol. Rev..

[30] Jawetz E, Gunnison JB, Coleman VR (1954). Observations on the mode of action of antibiotic synergism and antagonism. J. Gen. Microbiol..

[31] Cozens RM, Tuomanen E, Tosch W, Zak O, Suter J, Tomasz A (1986). Evaluation of the bactericidal activity of beta-lactam antibiotics on slowly growing bacteria cultured in the chemostat. Antimicrob. Agents Chemother..

[32] Ocampo PS, Lázár V, Papp B, Arnoldini M, Abel zur Wiesch P, Busa-Fekete R (2014). Antagonism between bacteriostatic and bactericidal antibiotics is prevalent. Antimicrob. Agents Chemother..

